# Extraction of Cell-free Dna from An Embryo-culture Medium Using Micro-scale Bio-reagents on Ewod

**DOI:** 10.1038/s41598-020-66779-z

**Published:** 2020-06-16

**Authors:** Anand Baby Alias, Cheng-En Chiang, Hong-Yuan Huang, Kai-Ti Lin, Pei-Jhen Lu, Yi-Wen Wang, Tzu-Hui Wu, Pei-Shin Jiang, Chien-An Chen, Da-Jeng Yao

**Affiliations:** 1 National Tsing Hua University, Department of Power mechanical Engineering, Institute of NanoEngineering and MicroSystems, Hsinchu, 30013 Taiwan; 2 Chang Gung Memorial Hospital, Department of Obstetrics and Gynecology, Taoyuan, 33305 Taiwan; 3 Chang Gung University and College of Medicine, Department of Obstetrics and Gynecology, Taoyuan, 33305 Taiwan; 4 National Tsing Hua University, College of Life Science, Hsinchu, 30013 Taiwan; 5 Industrial Technology Research Institute (ITRI), Biomedical Technology and Device Research Labs, Hsinchu, 31057 Taiwan

**Keywords:** Medical genomics, DNA sequencing

## Abstract

As scientific and technical knowledge advances, research on biomedical micro-electromechanical systems (bio-MEMS) is also developing towards lab-on-a-chip (LOC) devices. A digital microfluidic (DMF) system specialized for an electrowetting- on-dielectric (EWOD) mechanism is a promising technique for such point-of-care systems. EWOD microfluidic biochemical analytical systems provide applications over a broad range in the lab-on-a-chip field. In this report, we treated extraction of cell-free DNA (cf-DNA) at a small concentration from a mouse embryo culture medium (2.5 days & 3.5 days) with electro-wetting on a dielectric (EWOD) platform using bio-reagents of micro-scale quantity. For such extraction, we modified a conventional method of genomic-DNA (g-DNA) extraction using magnetic beads (MB). To prove that extraction of cf-DNA with EWOD was accomplished, as trials we extracted designed-DNA (obtained from Chang Gung Memorial Hospital (CGMH), Taiwan which shows properties similar to that of cf-DNA). Using that designed DNA, extraction with both conventional and EWOD methods has been performed; the mean percentage of extraction with both methods was calculated for a comparison. From the cycle threshold (C_t_) results with a quantitative polymerase chain reaction (q-PCR), the mean extraction percentages were obtained as 14.8 percent according to the conventional method and 23 percent with EWOD. These results show that DNA extraction with EWOD appears promising. The EWOD extraction involved voltage 100 V and frequency 2 kHz. From this analysis, we generated a protocol for an improved extraction percentage on a EWOD chip and performed cf-DNA extraction from an embryo-culture medium (KSOM medium) at 3.5 and 2.5 days. The mean weight obtained for EWOD-extracted cf-DNA is 0.33 fg from the 3.5-day sample and 31.95 fg from the 2.5-day sample. All these results will pave a new path towards a renowned lab-on-a-chip concept.

## Introduction

Genetic analysis is a complicated procedure involving three steps: (1) DNA extraction from raw biological samples, (2) sequence amplification with the real-time polymerase chain reaction (PCR) and (3) separation and selection of DNA for testing. The result of the first step can typically be used in diagnostic processes such as the detection of bacteria and viruses in the environment, to perform forensic analyses and to conduct a diagnosis of genetic disorder. An extraction of DNA can hence be considered to be one of the most essential parts of nucleic-acid testing. The entire procedure includes a series of reagent mixing and centrifugal separations, along with manual operations. DNA extraction conventionally utilizes a combination of physical processes to extract and to purify DNA from samples^1^. The nucleotides in DNA behave like magnets in the DNA replication and the shortening of the telomere^2^. Non-specific DNA binding means the binding of varied DNA molecules with approximately the same affinity to magnetic micro-particles, despite differences in the DNA sequence or the size of the various DNA molecules^3^. Here we exploit this magnetic property of DNA for its extraction. Magnetic particles are used in a wide range of medical and bio-related diagnostics carried out in microfluidic devices^4^. Magnetic Beads (MB) can even use as specific labels to concentrate or separate biological targets, e.g., cells, proteins, and DNA/RNA, from a sample^5^. A conventional method of g-DNA extraction using magnetic beads (MB) is explained in^1^. The ultimate resulting solution contained DNA that was the reaction product of PCR amplification. DNA can be detached from magnetic micro-particles with an elution buffer. Before this final separation of DNA that is bound to magnetic micro-particles, they must be washed with a suitable buffer solution, which has several characteristics. This solution must have a concentration of salt (i.e., ionic strength) sufficiently great that DNA bound to magnetic micro-particles does not elute from those micro-particles; a suitable salt concentration is greater than about 1.0 M and preferably about 5.0 M. A buffer solution is chosen so that impurities that are bound to DNA or micro-particles are dissolved. As for the expected impurities, the pH, solute composition and concentration of the buffer solution can be varied. A suitable wash solution includes SSC (0.5 × 5), ammonium sulfate (100 mM), Tris (400 mM, pH 9.25), MgCl^2^ (25 mM), bovine serum albumine (BSA, 1 percentage) and NaCl (5 M). The magnetic micro-particles with bound DNA can be washed with more than one wash buffer solution; the number of such washings is preferably limited to two or three to minimize the loss of the bound DNA. The yields of DNA after eluting with an elution buffer can be 80 percentages when the micro-particles are used in excess^3^.

Much test sample and reagent and an enormous working laboratory or spaces are required for DNA extraction with a conventional method. To overcome these limitations, we applied a lab-on-a-chip (LOC), which is a device that integrates one or several laboratory functions on a single integrated circuit (commonly called a chip) of size only mm^2^ to a few cm^2^ to achieve automation and high-throughput screening. A LOC can operate with minute fluid volumes to less than the mL range. LOC devices are a subset of micro-electromechanical systems (MEMS) that might use micro-fluidics, physics and manipulation for the study of fluids in minute amounts. A digital microfluidic system (DMF) can be utilized and adapted towards a LOC. A DMF controls and manipulates fluids as discrete droplets on an array of independent electrodes on a microchip. The droplets are individually manipulated on applying signals to these electrodes^1^.

EWOD is a DMF technique that has been recently introduced as a method towards LOC systems. Droplets of unit volume are manipulated along electrode arrays, allowing a microfluidic function to be reduced to a set of basic operations^6^. The droplets are initially placed in reservoir electrode(s), then split into multiple droplets and mixed with others for reactions of various types^1^. Microfluidic operations with EWOD actuation feature precise droplet actuation, decreased risk of contamination, decreased volume of reagents, and increased efficiency of reagent mixing and decreased duration of reaction. Typical microfluidic systems process liquids on pumping them in micro-channels. An advanced EWOD-based DMF system generates and manipulates droplets in a thin space on a EWOD chip with electric signals from the control PC for electrically activating the electrodes by a voltage amplifier. Thus, there is no need of pump or valve as in typical microfluidic systems. This simplicity and portability makes a EWOD-based DMF system widely popular in biomedical or chemical fields as a powerful platform for sample preparation. With this method, we have the possibility to perform on-chip all fundamental fluidic operations (droplet dispensing from a reservoir, transport, merging, mixing and splitting) using an array of electrodes. Various applications of EWOD in the biomedical field are reported^7^. Here we exploit a EWOD method for the extraction of cell-free DNA from an embryo-culture medium.

The result of the DNA extraction can be analyzed with a quantitative polymerase chain reaction (q-PCR), which is a supremely sensitive method for the detection of target DNA in minute amounts. The advantages of q-PCR analysis are its rapid analysis, high sensitivity and unparalleled specificity. Two important advantages are that the thermal cycler and the temperature cycling parameters are the same as for the end-point PCR^8^, but the exponential nature of DNA amplification is prone to burden experimental data with significant standard error due to the inherent variations of amplification efficiency from tube to tube^9^. A standard curve (calibration curve) is a graphical analysis of a type used as a quantitative research technique in which multiple samples with known properties are measured and graphed, from which the same properties are deduced for unknown samples on interpolation in the graph^10^. Here we used a standard curve analysis to achieve a quantitative analysis of unknown cf-DNA samples.

## Results

**Figure 1:**
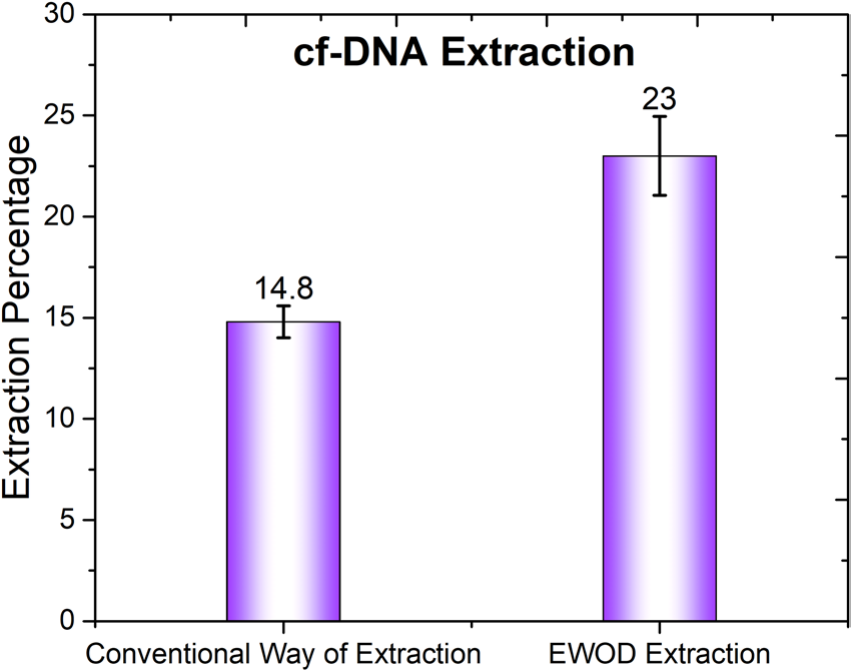
Comparison between average extraction percentage for conventional and EWOD methods of cf-DNA extraction.

EWOD Extraction to find the efficiency of cf-DNA extraction procedure in EWOD platform, we initially performed extraction of designed-DNA (obtained from CGMH, Taiwan which shows properties similar to that of cf-DNA) in conventional way. On repeating experimental analysis we found that the conventional extraction yielded an extraction 14.8 percentages; later we tried the same on a EWOD system, for which the extraction yielded 23 percentages. Figure 1 shows a comparison of results between conventional and EWOD methods of cf-DNA extraction. The equation to find the percentage of extraction is E (percentage) = [1/2^[Ct(B)-Ct(A)]^] * 100 in which E (percentage) = Extraction Percentage, C_t_(B) = template C_t_, C_t_(A) = C_t_ control.

From the result of the comparison, the DNA extraction with a EWOD system looks promising.

**Figure 2:**
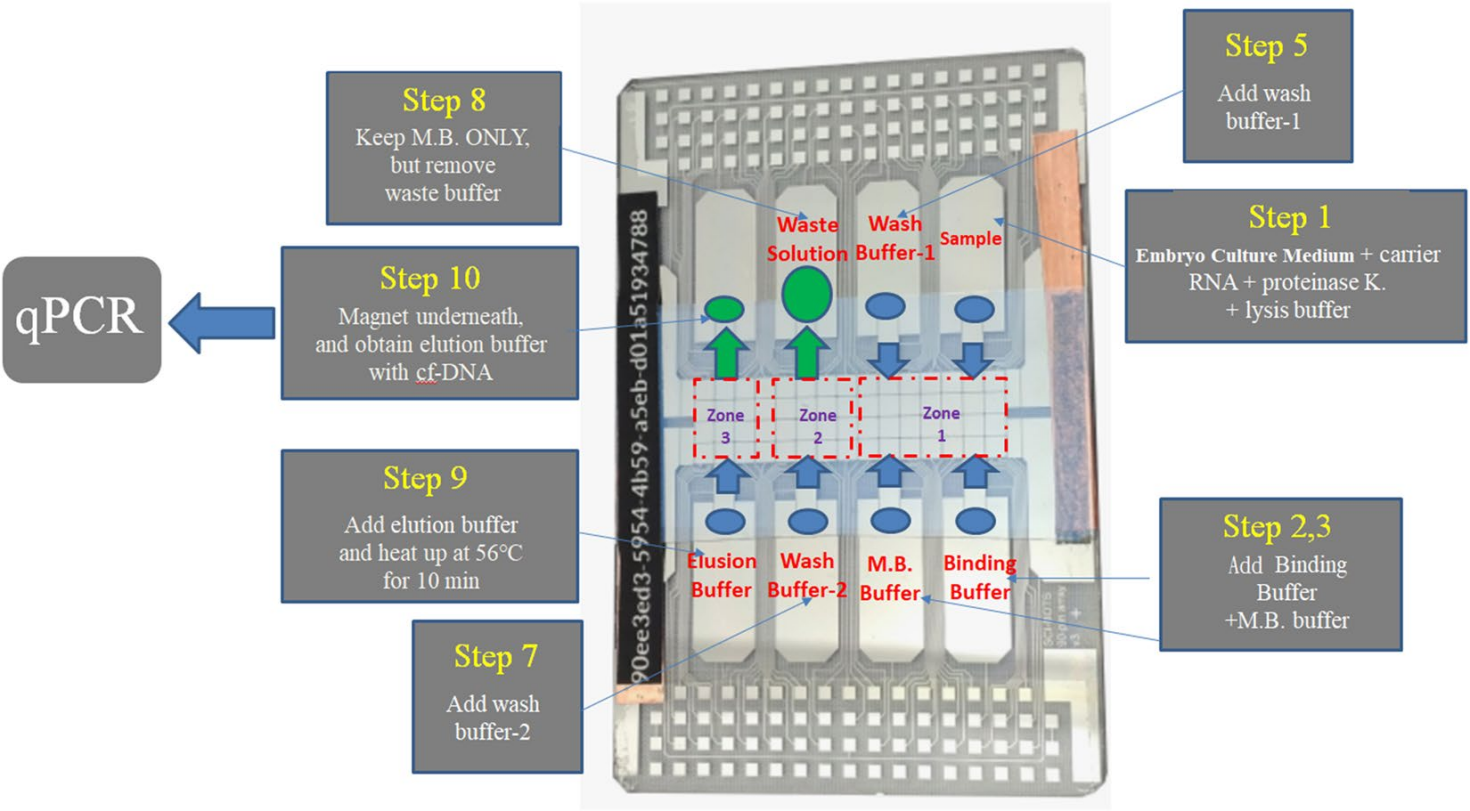
Placement and steps of bio-reagents used for cf-DNA extraction with EWOD.

**Figure 3:**
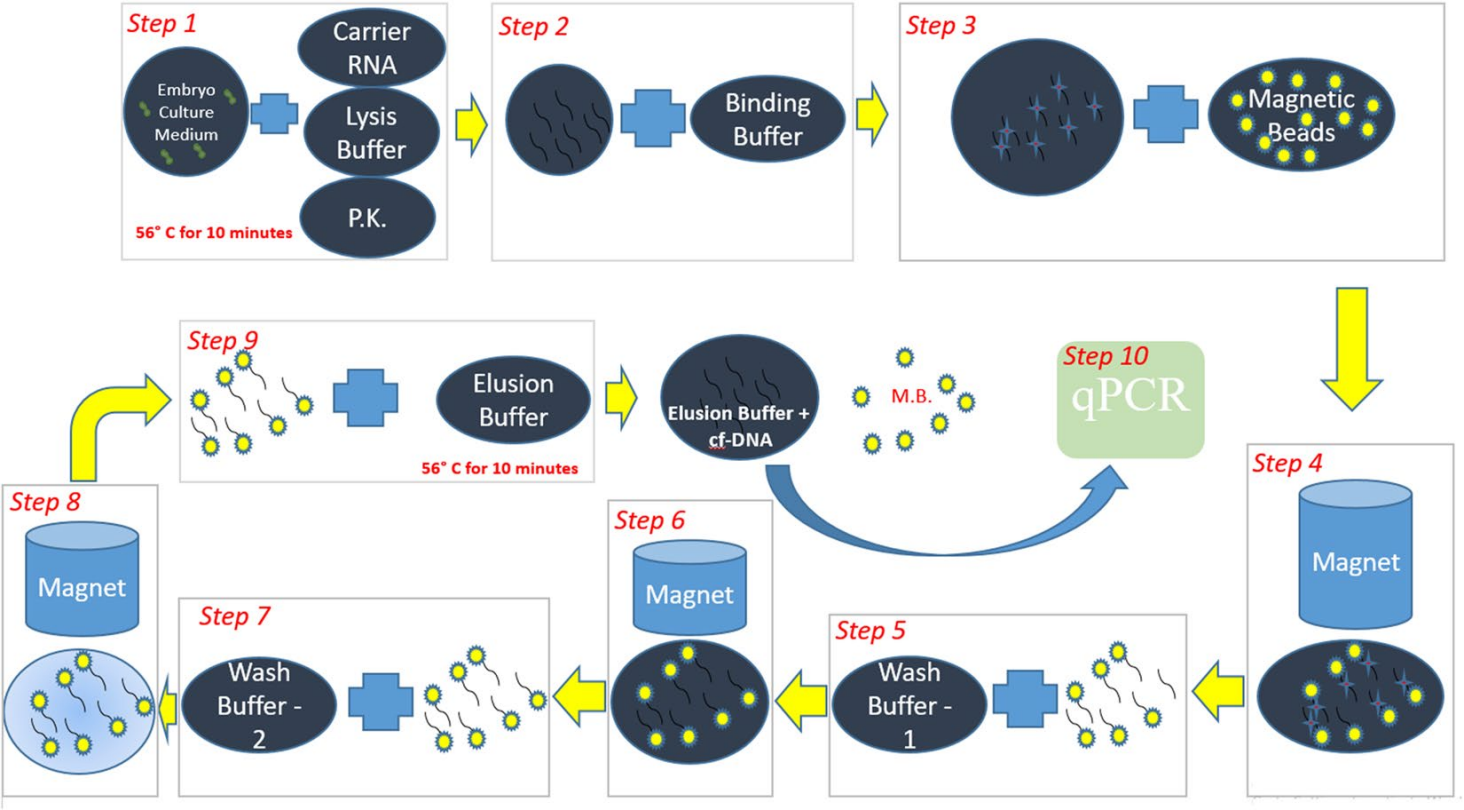
Typical Procedures of cf-DNA extraction with magnetic beads.

The minimum voltage required to generate and transport bio-buffers is 100 V_pp_ at frequency 2 kHz, except wash buffers 1 and 2 required only a minimum 80 V_pp_ at 2 kHz. At 100 V for wash buffers, bubble formation occurs which affects the mixing efficiency. Fig. 2 represents the placement and adapted steps of various bio-buffers on a EWOD chip for a comparison with the steps in Fig. 3 (typical way to extract cf-DNA using magnetic beads). The electrodes in the chip (Fig. 2) can be classified into 3 sets. This includes the reservoir electrode, generation electrode and transportation electrode. To the reservoir electrode, the samples are initially placed. Then using generation electrode (very next to reservoir electrode) and transportation electrode(s) the required amount of droplet is generated. Moreover to avoid contamination due to overlapping steps, we split the entire chip virtually into three zones as shown in Fig. 2. Transport electrodes in zone 1 served to mix the sample (embryo culture medium, carrier RNA, P.K), binding buffer and magnetic beads. Then after separation using a magnet, the MB gets washed with wash buffer 1. After that first washing, the MB was separated by a magnet. Zone 2 is used for further cleaning with wash buffer 2. After this cleaning, the separation of the MB from wash buffer 2 was carried out with the magnet. Zone 3 region was used exclusively for the final eluting step. We avoided contamination to a maximum extent.

Besides higher extraction percentage, EWOD method is more cost effective and eco-friendly than conventional method. By using EWOD, we can avoid using supplementary requirements like Eppendorf (In conventional method, usually we used 5 nos. for one-time extraction) and Eppendorf pins (more than 15 numbers) in a maximum extent. The result of this leads to minimization of biological debris which is one of the prime issues faced by nature. Moreover, the EWOD extraction is more user friendly than conventional extraction.

**Figure 4:**
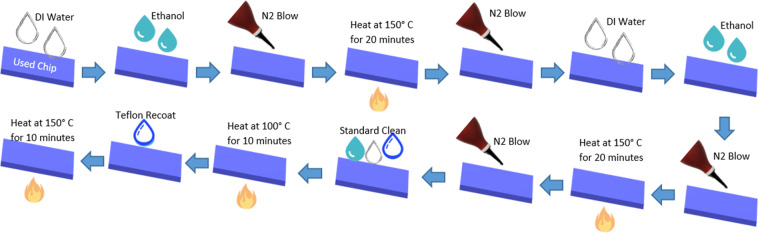
Steps for chip cleaning to remove residual cf-DNA from used EWOD chip.

Chip Cleaning On investigation to find any DNA remaining after extraction using EWOD chip, we ran DD water through the entire electrodes after extraction. This DD water had undergone q-PCR analysis; the result showed the presence of DNA still remains on the chip, so we had to clean the chip properly before reusing it for the next extraction. Figure 4 represents the steps involved in cleaning the EWOD chip. Here the used chip initially undergone ethanol bath followed by DI water clean and nitrogen blow. Then heat the chip at 150 degree C for 20 minutes. Repeat the process and then perform standard clean (Clean with Acetone, IPA and DI water). Then heat the chip at 100 degree C for 10 minutes. Now do Teflon coating at 3000 rpm for 30 seconds followed by baking the chip at 150 degree C for 10 minutes. After the whole cleaning as explained, we again run DD water along the entire electrodes; the result showed no C_t_ value, which meant that the chip contained no DNA residue. Table 1 gives some results after chip cleaning.

**Table 1:** Result after performing chip cleaning.

Sample No.	Obtained C_t_ value for cf-DNA Extraction	C_t_ value for unbounded DNA	C_t_ value after chip cleaning
1	27.28	31.76	Nil
2	27.13	28.32	Nil
3	27.28	31.31	Nil
4	25.37	Nil	Nil
5	30.66	30.87	Nil
6	29.08	31.45	Nil
7	29.29	30.92	Nil
8	30.4	Nil	Nil

### q-PCR analysis

**Figure 5:**
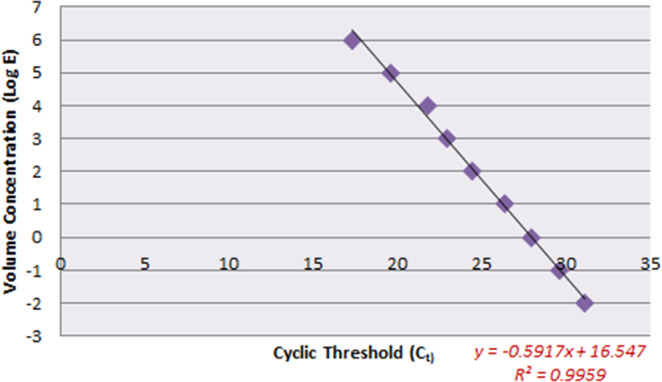
Standard curve plot. Abscissal axis shows the C_t_ values obtained during q-PCR for various concentrations (ordinate axis) of samples.

**Figure 6:**
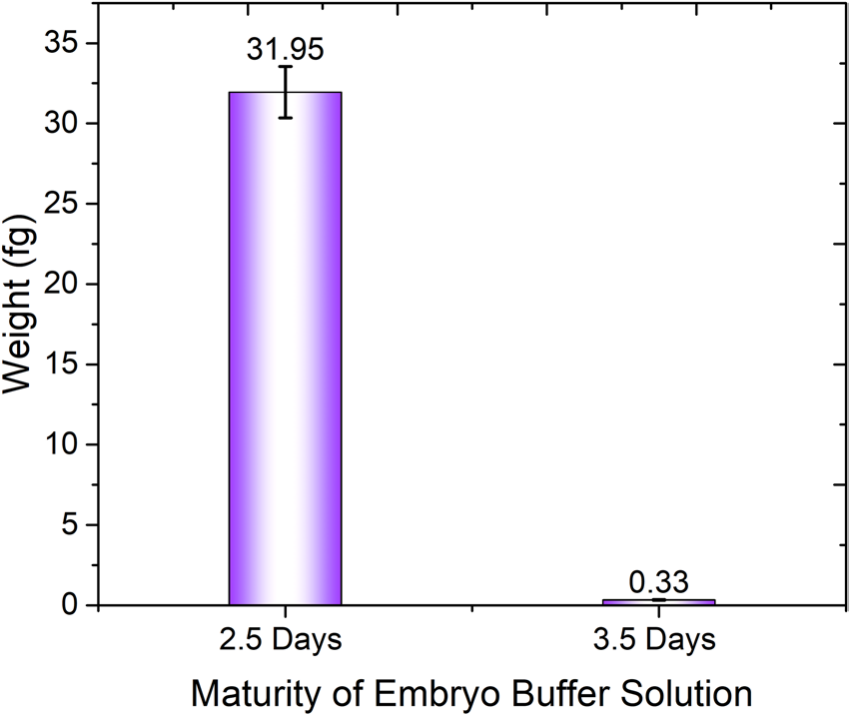
Mean weight/fg of EWOD-extracted cf-DNA from an embryo-culture medium (2.5 days & 3.5 days).

The result of elution was given for a q-PCR analysis. For this q-PCR analysis, a final elution buffer (1 microliter) after extraction was mixed with master-mix (5 microliter), primer-forward (0.2 microliter), primer-reverse (0.2 microliter) and DD- water (3.6 microliter) to form 10 microliter altogether. The result of each q-PCR gave a particular threshold cycle (C_t_) value that could be further used for a quantitative analysis of the given sample. To calculate the concentration of each extracted elution result and toward the unknown weight of the samples, we plotted a standard curve with a control sample from a mother mouse having 7 ng/microliter. Volume concentrations that varied from 1 ng to 0.01 fg for respective equivalent dilution values were prepared for the standard curve as shown in Table 2. Figure 5 shows the obtained standard curve plot, for which R^2^ = 0.9959; the obtained plot has hence great precision to calculate an unknown concentration. From the equation obtained from the standard curve plot, we calculated the concentration of an unknown sample along with the obtained weight of every sample. We undertook cf-DNA extraction on EWOD of an embryo-culture medium from 3.5 and 2.5 days. Table 3 shows the weight of some extracted cf-DNA from different embryo-culture mediums at both 3.5 and 2.5 days. Figure 6 gives the mean weight of EWOD extracted cf-DNA from the embryo-culture medium at both 3.5 and 2.5 days. As we performed three times of final eluting, we need to multiply the final mean result by three. Hence the mean masses for cf-DNA are 0.33 fg from the sample at 3.5 days and 31.95 fg from the sample at 2.5 days.

**Table 2:** Standard curve for quantitative analysis of unknown cf-DNA.

Concentration of control sample	E = Equivalent Dilution Value/fg	Log (E)
1 ng	1000000	6
0.1 ng	100000	5
0.01 ng	10000	4
1 pg	1000	3
0.1 pg	100	2
0.01 pg	10	1
1 fg	1	0
0.1 fg	0.1	−1
0.01 fg	0.01	−2

**Table 3:** C_t_ value obtained for cf-DNA extractions and calculated masses.

Sl. No.	Maturity of embryo- culture medium	C_t_ value	Calculated concentration	Final obtained mass/fg
1	2.5 days	27.28	0.4054	7.6
2	2.5 days	27.13	0.4942	9.3
3	2.5 days	27.28	0.4054	7.6
4	2.5 days	25.37	1.5356	102.9
5	2.5 days	25.67	1.3581	68.4
6	3.5 days	30.66	−1.5945	0.076
7	3.5 days	29.63	−0.9851	0.310
8	3.5 days	29.08	−0.6596	0.656
9	3.5 days	29.29	−0.7839	0.493
10	3.5 days	30.4	−1.4407	0.108

The sample we used for DNA extraction is ‘embryo culture medium (KSOM)’. In 2.5 days, the embryo become in 4 cell stage and in 3.5 days it develops to 8 cell or morula stage. So the 2.5 days sample has patrilineal and maternal DNA contamination with fetal DNA in embryo development stage. But during the next level (3.5 days), the patrilineal and maternal DNA level is reduced because of meiosis. Thus the cf-DNA in 2.5 days will be more when compared with 3.5 days.

## Discussion

This paper reports the extraction of designed DNA and cell-free DNA (cf-DNA) at small concentrations on an electro-wetting on dielectric (EWOD) platform using bio-reagents of micro-scale quantity. A refinement of the conventional genomic-DNA (g-DNA) extraction method for cf-DNA extraction is explained and implemented for an improved extraction percentage. The extraction of designed-DNA was performed with both conventional and EWOD methods; the mean extraction percentage was calculated for comparison. Cycle threshold (C_t_) results were obtained with a quantitative polymerase chain reaction (q-PCR); the mean extraction percentages obtained were 14.8 percentages for the conventional method and 23 percentages for the EWOD at 100 V, 2 kHz. This result shows that DNA extraction appears promising with the EWOD system. In DMF platform, once the sample has put over the chip, there is no more other direct external interference towards the sample mixture. At the same time, in conventional way, we need to transfer the mixture to different eppendorf and need to use more than 10 eppendorf pins (for mixing and sample/residue transferring) during the extraction process, which all affects the final result. Moreover the mixing of samples can be performed well in EWOD than conventional way. All these favor EWOD to yield more cf-DNA. We proceeded to perform cf-DNA extraction from an embryo-culture medium at 3.5 and 2.5 days (in KSOM medium). The mean masses obtained for EWOD extracted cf-DNA are 0.33 fg from the sample at 3.5 days and 31.95 fg from the sample at 2.5 days. All these results will pave a new path towards a renowned lab-on-a-chip concept.

## Methods

### Embryo-culture Medium and Cell-free DNA

Cell-free DNA (or cf-DNA) refers to all non-encapsulated DNA in the blood stream. The cf-DNA comprises nucleic acid fragments that enter the bloodstream during apoptosis or necrosis^11^. Theoretically, circulating DNA is released mainly from degrading cells after endonucleases that cut chromatin into basic nucleosomal elements^12^. The amount of free circulating DNA in a serum is reported to be six times that in plasma^13^. Circulating free DNA has been intensively investigated as a biomarker for malignancy and other diseases^13^. Here we report a method for the EWOD extraction of cell-free DNA from an embryo-culture medium used for development of a mouse embryo; the culture medium used is KSOM medium. The conditioned mouse embryo at the two-cell stage was placed in a culture medium of KSOM medium for development. The development of the mouse embryo *in vitro* from a two-cell stage to a four-cell stage (blastocyst stage) is explained elsewhere^14^. The advantage of ovoil in a cultural medium is explained in^15^. The KSOM medium helps to maintain the preferred environmental condition for an embryo development. Fertilization *in vitro* (IVF) using ovoil along with cultural medium is explained in^16^. We used a mouse-embryo-culture medium and designed DNA from Chang Gung Memorial Hospital (CGMH), Linkou, Taiwan. For introductory tests and experiments, we used designed DNA, which shows properties similar to that of cf-DNA.

### Proposed cf-DNA extraction with a conventional method

The typical way to extract cf-DNA using magnetic beads is shown in Fig. 3. The embryo-culture medium was incubated at −80 °C. The bio-buffers used for cf-DNA extraction included carrier RNA, Proteinase k (P.K.), lysis buffer, binding buffer, magnetic beads, wash buffer-1, wash buffer-2 and elution buffer. The function of each bio-buffer is given in Table 4. All these bio-buffers used to extract cf-DNA were provided by Industrial Technology Research Institute (ITRI), Taiwan. From several experiments using designed DNA, we found that the residue from step- 4 results and MB in step-9 results (Fig. 3) still have DNA. The step-4 residue contains on average 1.75 percentages DNA; regarding step 9, after one-time eluting, the MB contains on average 3.2 percentages DNA. After performing a second elution on this MB, it still contains an average 0.7 percentage DNA, so we performed a further eluting step on this MB. The residue MB then had on average only 0.3 percentage DNA, which we hence ignored. We thus finalized to perform eluting three times. Figure 3 has hence been refined. The modified steps from Fig. 3 for cf-DNA extraction from an embryo-culture medium using the above-mentioned bio-buffers were as follows. In step 1, a quantity (1 microliter) of sample (embryo-culture medium) was collected and mixed with carrier RNA, P.K. and lysis buffer. The mixed solution was heated at 56 °C for 10 min. In step 2, this mixture was then mixed with a binding buffer and flowed by magnetic beads mixing (step 3); the binding buffer enhanced the attachment of DNA participles to the magnetic beads. In step 4, the magnetic beads were separated from the other solution using a magnet. To test whether the separated solution had still some cf-DNA, we added more magnetic beads to the separated solution (residue), before separation with a magnet. From several experiments, we found that this residual solution that was separated from the magnetic beads still had some DNA remaining. To improve the rate of extraction, after step 4, we added more magnetic beads to the residual solution after separation via a magnetic field. Then separate this MB by magnet and combined both sets of magnetic beads and proceeded to the next procedure. These magnetic beads that had cf-DNA must be cleaned before detachment. For this purpose, (in step 5–8), we used wash buffer 1 and wash buffer 2 for washing twice. After each washing step, the separation of magnetic beads from the wash buffer solution was effected with a magnet. After washing, the DNA must be detached from the magnetic beads, for which, in step 9, an elution buffer is used. After adding an elution buffer to the magnetic beads, the solution was heated at 56 °C for 10 min. This heating step enhanced the detachment. After heating, the elution buffer having DNA was separated from the magnetic beads with a magnet. After this step, we had magnetic beads as a residue and an elution buffer as a final result having cf-DNA. To ensure that all DNA was detached from the magnetic beads, we added more elution buffer and repeated step 9; the result showed that the DNA was not fully detached from the magnetic beads. To maximize the yield, after wash-buffer cleaning, we repeated step 9 three times. The final elution result was collected in an eppendorf tube and incubated at −80 °C for q-PCR analysis (Step 10). All methods were performed in accordance with the relevant guidelines and regulations of ITRI and CGMH, Taiwan.

**Table 4:** Functions of different bio-buffers for DNA extraction.

Bio buffers	Functions
Lysis buffer	To break down the cell walls completely to release the DNA from the nucleus
Proteinase K	To hydrolyse the histones such that the DNA in WBC was available for subsequent reactions
Binding buffer	To provide an appropriate ion condition for DNA to bind with beads
Wash buffer 1,2	To clean MB
Elution buffer	To elute the DNA from the MB

### Adaptation of cf-DNA extraction towards an EWOD system

**Figure 7:**
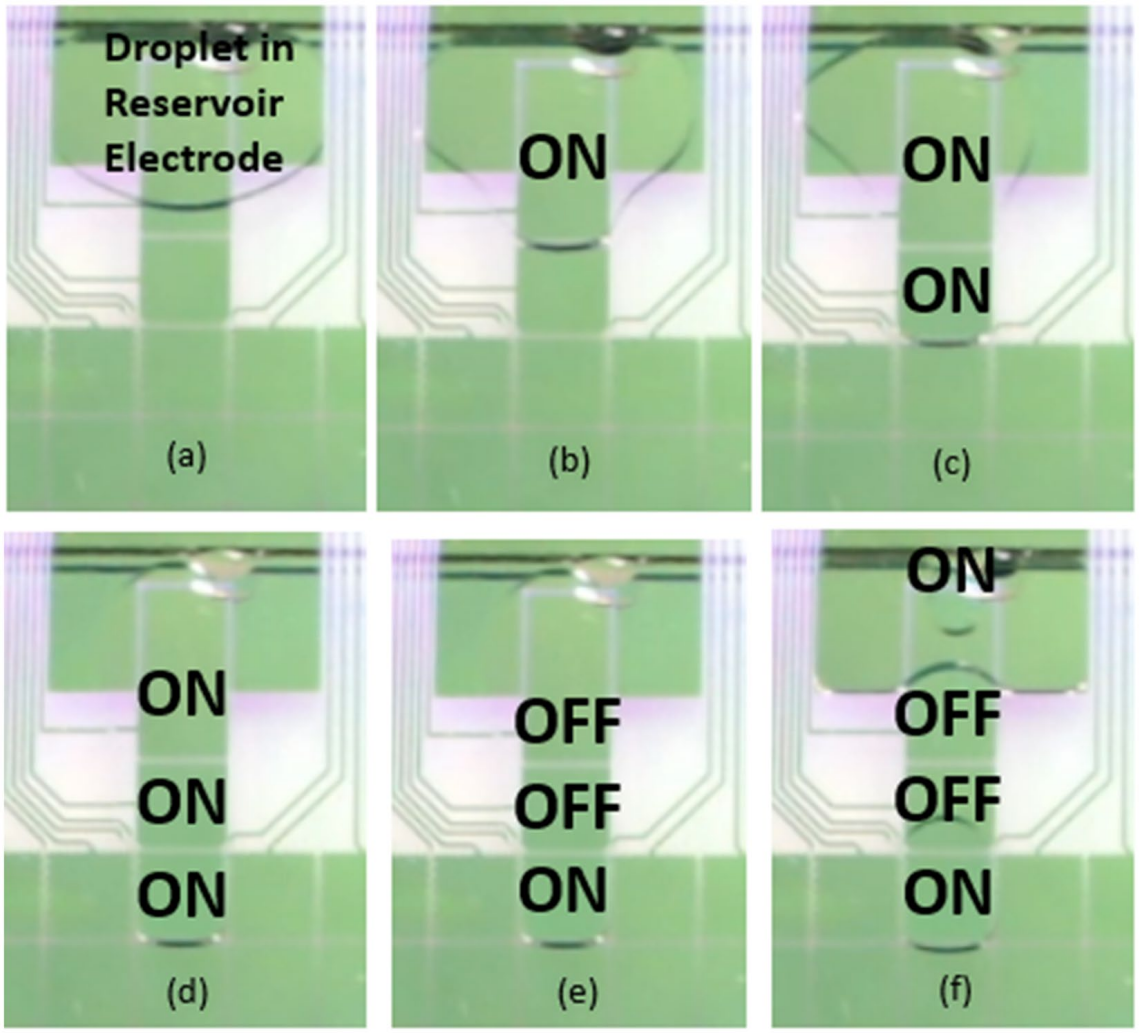
Droplet Generation. (**a**) Reagent was placed on the reservoir electrode, (**b**) the generation electrode was turned ON, (**c**,**d**) Adjacent transport electrodes (based on droplet size requirement) the electrodes were turned ON as shown in figure (here two transportation electrodes turned ON), (**e**) Turn OFF all the transportation electrodes (except the end one) (**d**) Turn ON the reservoir electrode. Thus a droplet is generated at the end transportation electrode which is in ON state.

The raw physiological samples have been introduced onto the chip and further processed by lysing blood cells and extracting DNA^17^. Electrowetting has become a widely used tool to manipulate liquids in tiny amounts on surfaces. EWOD-driven devices generate less Joule heating and can also enable excellent control of a fluid flow as they are reconfigured simply on reprogramming the sequence of potentials applied to the electrodes. The chip system had basically a top plate and a bottom plate; the droplet was placed in the channel gap between the two parallel plates. The top plate was an indium-tin-oxide (ITO) glass substrate. The bottom plate was patterned with electrode arrays covered with dielectric materials. To manage a EWOD system, there are three important design parameters, namely the channel gap, the electrode pitch and the electrode gap. This channel gap refers to the gap between the top and bottom plates, which is created on placing a double-sided tape between the parallel plates. The electrode pitch refers to the distance from one electrode to the other; the electrode gap refers to the gap between the two electrodes^18^. The entire conventional process to extract cf-DNA explained above was implemented in a EWOD system. The EWOD chip can basically be categorized into three types, which includes (a) a reservoir electrode, (b) a generation electrode and (c) a transport electrode. All bio-buffer reagents used for cf-DNA extraction were kept in separate reservoir electrodes. Droplets (of microliter size) of bio-buffers were generated using a generation electrode. Mixing, transport and separation steps were undertaken with transport electrodes. For a faster mixing time, a greater number of electrodes can be used while operating at higher frequencies. Mixing in electrowetting systems can be greatly accelerated by the active manipulation of generated droplets^19^. By applying a sequence of potentials to adjacent electrodes on an array, aqueous droplets can be made to transmit across EWOD surface^20^. We used ‘Dropbot Model: DB3–120’ (Sci-bots Company, Canada) as a EWOD platform to implement the extraction. The details of a similar system are given in^21^. The chip has basically a top plate and a bottom plate; the gap between these plates is 180 micrometers; the dimensions of the electrode are 2.25 mm × 2.25 mm. Each transport electrode can hold 1 microliter of bio-buffer. However, it strictly depends on the number of transportation electrodes used for activation (ON/OFF). An amount of 1 microliter is generated when we activate one transportation electrode along with the generation electrode. In Fig. 7, two transportation electrodes are used along with the generation electrode for droplet generation. Hence the volume of droplet is around 1.8 microliter. Figure 7 shows the generation of the bio-buffers on a EWOD chip. After performing the entire extraction on a EWOD chip, the final elution buffer was collected to undergo a q-PCR analysis (system qTOWER3G). The reagents for q-PCR analysis included a master-mix, a primer-forward, and a primer-reverse and distilled deionized (DD) water. The master mix used, KM4100, was sensitive to light. All these were kept at −20 °C.

## Supplementary information

DNA extraction.
